# Using health administrative data to model associations and predict hospital admissions and length of stay for people with eating disorders

**DOI:** 10.1186/s12888-023-04688-x

**Published:** 2023-05-10

**Authors:** Marcellinus Kim, Matthew Holton, Arianne Sweeting, Eyza Koreshe, Kevin McGeechan, Jane Miskovic-Wheatley

**Affiliations:** 1 The University of Sydney, Sydney, Australia; 2 Sydney Local Health District, New South Wales Health, Sydney, Australia; 3 The University of Sydney and Sydney Local Health District. Royal Prince Alfred Hospital, Sydney, NSW Australia

**Keywords:** Eating disorders, Models, Emergency service, Length of Stay, Predict

## Abstract

**Background:**

Eating disorders are serious mental illnesses requiring a whole of health approach. Routinely collected health administrative data has clinical utility in describing associations and predicting health outcome measures. This study aims to develop models to assess the clinical utility of health administrative data in adult eating disorder emergency presentations and length of stay.

**Methods:**

Retrospective cohort study on health administrative data in adults with eating disorders from 2014 to 2020 in Sydney Local Health District. Emergency and admitted patient data were collected with all clinically important variables available. Multivariable regression models were analysed to explore associations and to predict admissions and length of stay.

**Results:**

Emergency department modelling describes some clinically important associations such as decreased odds of admission for patients with Bulimia Nervosa compared to Anorexia Nervosa (Odds Ratio [OR] 0.31, 95% Confidence Interval [95%CI] 0.10 to 0.95; p = 0.04). Admitted data included more predictors and therefore further significant associations including an average of 0.96 days increase in length of stay for each additional count of diagnosis/comorbidities (95% Confidence Interval [95% CI] 0.37 to 1.55; p = 0.001) with a valid prediction model (R^2^ = 0.56).

**Conclusions:**

Health administrative data has clinical utility in adult eating disorders with valid exploratory and predictive models describing associations and predicting admissions and length of stay. Utilising health administrative data this way is an efficient process for assessing impacts of multiple factors on patient care and predicting health care outcomes.

**Supplementary Information:**

The online version contains supplementary material available at 10.1186/s12888-023-04688-x.

## Introduction

Eating disorders (ED) are serious mental illnesses with a complex range of medical complications and mental health comorbidities [1]. Adults with ED can present to emergency departments with acute medical instability and/or acute behavioural and psychosocial deterioration. Liaison and interface between medical and mental health care, to treat aspects of the illness and prevent mortality, is essential [2]. All EDs have an elevated mortality risk, highest with AN [3]. It is estimated that over a million Australians are living with ED, with reported incidence ranging from 4% [4] to 16.3% [5]. Furthermore, ED are frequently chronic or relapsing [6], with an average duration of illness of 15 years [4]. Therefore ED “burden of disease” and health system expenditure are high [4], exacerbated by an increasing trend of people with ED accessing the health system [7]. Furthermore, ED are complex with significant clinical heterogeneity and may be a multidimensional disorder which includes several subtypes with different neurobiological underpinnings, and often associated with several other psychiatric disorders as well as suicide risk [8, 9].

There is limited evidence for highly efficacious interventions and optimal treatment options and pathways of care [10, 11]. Health care outcomes such as length of stay and mode of separation, which is the way in which a person leaves the hospital or service setting, are widely reported and used to guide services and clinicians on clinical decision, opportunities for improving clinical care provision and resource allocation. Whilst there is a growing body of literature in ED, there remains a lack of evidence and studies on defining clear and consistent measures for outcome, severity, remission and recovery [12].

Hospital data from New South Wales (NSW) in Australia shows a progressive, greater than 2-fold increase in emergency department triage and hospital admissions for people with ED since 2011 [13]. Furthermore, the impacts from the COVID-19 pandemic are apparent in this data with a disproportionate increase in admissions to hospital in particular for under 16-year-olds since the beginning of the pandemic [13]. Recent studies confirm this ongoing trend, on the impact of COVID-19 and associated public health response on people with ED and other mental health conditions [14, 15]. There is a clear need for effective and efficient use of the current services and resources available to provide integrated and optimal care and support for people with ED, their families and carers.

In 2013, the NSW Service Plan for People with Eating Disorders was established, providing a framework for multidisciplinary health professionals to support the delivery of treatment and care for people with or at risk of developing an eating disorder. This guides the Sydney Local Health District (SLHD) Eating Disorder Service Plan (2019–2024) [16] aimed at developing and integrating mental health and medical services across the district. SLHD has an established local and tertiary eating disorder service, providing statewide outpatient and inpatient treatment. This follows a case formulation driven framework, determining the level of support required for the person at the point of care. People with ED in SLHD who present with medical instability are assessed and triaged via the emergency department and admitted to a medical ward. Following a medical admission, a patient may be admitted to the Peter Beumont Unit (Specialist ED Ward) located at Royal Prince Alfred Hospital (RPA) or attend outpatient eating disorder services or other local services determined by a multidisciplinary team. Adults with ED may also be admitted to general Mental Health wards in SLHD.

In NSW Health, there is routinely collected health administrative data, which could be utilised to examine health care outcomes, namely, the NSW Emergency Department Data Collection (EDDC) recording all emergency department presentations and NSW Admitted Patient Data Collection (APDC) recording inpatient separations (i.e. referrals post-discharge). There are limitations with health administrative data in relation to varied accuracy, validity, and coding. However, the main advantages of utilising administrative data are efficiency, representative of all admitted patients, readily available and routinely collected with large quantities and breadth of data with numerous variables collected.

While it is well established that health administrative data can be useful in predicting and identifying associated factors on health system outcomes and care needs [17–20], this has yet to be explored in ED. The overall aim of the study is to assess the clinical utility of routinely collected health administrative data. To do this, initial exploratory multivariable regression models will be analysed to identify and describe clinically plausible factors associated with admissions in emergency department and inpatient length of stay for admitted patients to hospital. Furthermore, prediction models will also be developed and validated on these health care outcome measures of patients with ED.

## Method

This is a retrospective cohort study on routinely collected health administrative data in people ≥ 16 years of age with ED. The search criteria included all consecutively selected ED primary or other diagnosis in emergency or admissions to the hospitals in SLHD from 2014 to 2020. ED diagnosis is in accordance with ICD-10 codes for admitted data or SNOMED codes with confirmed ED diagnosis in emergency department data. SNOMED codes specifies Anorexia Nervosa and Bulimia Nervosa but describes other eating disorders diagnoses as generalized “Eating Disorder” and is descriptive in nature. The years 2014 to 2020 was chosen to capture the time from stage 1 NSW Service Plan for People with Eating Disorders. The emergency department data had additional data from 2011 so this is also included. As this data was extracted in August 2021, it allowed for inclusion of all patients admitted in 2020 including people discharged in 2021.

A data variables list was developed from the data dictionaries, NSW Emergency Department Data Collection (EDDC) and NSW Admitted Patient Data Collection (APDC) datasets that are available on the CHeReL website [21]. CHeReL is managed by the NSW Ministry of Health and its function is to carry out linkage of health-related data and provide a mechanism of access to these linked data. This information and search criteria were provided to SLHD data custodians for data extraction from SLHD electronic medical records (Cerner). A Statutory Query Language (SQL) search was used to extract SLHD emergency department data (EDD) and SLHD admitted patient data (APD). The investigators then conducted data and statistical quality assurance process’ including data cleaning and manipulation prior to analysis.

This study was approved by the Human Ethics Review Committee (Royal Prince Alfred Hospital Zone) of SLHD, Australia, protocol number X21-0059.

### Outcome Measures

Mode of separation is the way in which a person leaves the hospital or service setting, and this is utilised as the main outcome measure for a patient admitted or not admitted from the EDD. For the APD, the outcome measure is defined as an episode of care with length of stay to be the total length of stay from when a person is admitted to when they were discharged. It is determined that this would be the most appropriate and clinically relevant definition for this study to explore factors that impact on the total duration of a patient’s whole episode of care.

### Explanatory/Predictor Variables

All variables available from routinely collected health data were considered for inclusion in the analysis. Initial data collection from the EDD and APD were reduced to clinically significant variables based on consideration of descriptive statistics and clinical relevance/importance. The APD included diagnostic variables so initially, broad diagnostic groupings or index scores such as the Charlson Comorbidities Index and Elixahauser Index were considered, however from careful exploration of the weighting and the evidence [22, 23] in the clinical context of ED, it was decided that broad weighted groupings and indexing would not be clinically appropriate or meaningful. Therefore, diagnoses were grouped into diagnostic categories. Each diagnostic category was revised multiple times taking into account descriptive statistics, ICD-10-AM categorisations and clinical context/meaning. The final diagnostic groupings consisted of 10 mental health comorbidities and 9 physical health comorbidities. (Supplementary 1)

### Statistical Analysis

Data was analysed using STATA Version 15 (StataCorp, College Station, TX, USA). Alpha of 0.05 was considered statistically significant. A full model for exploratory purposes as well as a main effects model for prediction were developed for both the EDD with the binary outcome of admission or discharge and for the APD with length of stay as outcome. A multivariable logistic regression model was chosen for modelling the odds of an admission from emergency department from the EDD and a multivariable linear regression model was chosen for modelling length of stay from the APD. Due to skewness of the APD data, further sensitivity analyses were conducted using a multivariable negative binomial regression model.

### Model Building Strategy

For both multivariable initial exploratory models, variable selection was based on clinical context, appropriateness and importance of categorisation and transformation of covariates in the context of statistical analyses. For both final prediction models, the model building strategy followed the “Purposeful Selection of Covariates” as outlined by Hosmer et al. [24] The general principle of the model building strategy involved careful exploration and selection of each of the covariates based on clinical relevance and importance followed by statistical analyses in a cyclical iterative process. All potential interactions were considered clinically and then statistical analyses of potential interactions were explored. Investigators representing different disciplines with expertise in their area reviewed each step of this process, until a consensus was reached.

Validation of the final main effects models for prediction included tests on residuals, heteroscedasticity, goodness of fit tests, variation inflation factors testing for multicollinearity and exploration of outliers. A regular bootstrap procedure was followed for internal validation of the predictions in both prediction models. A regular bootstrap including 100% of the sample, with 1000 repetitions was used for internal validation [25, 26].

## Results

### Patient Characteristics (Modelling Admissions in Emergency)

There were 228 presentations from the EDD identified as diagnosed with ED between 2011 and 2020. The mean age is 30.0 years (SD = 14.7, range 16 to 87). Referrals were from self, family or friends (70.2%) or ‘other’ health, aged care and community services (29.8%). The most presentations to emergency department occurred in 2019, with almost half of presentations identified with a diagnosis of Anorexia Nervosa. Most patients were female (92.1%) and presented to RPA (84.6%) compared to other hospitals in SLHD. Patient characteristics are available in Table 1.

**Table 1:** Characteristics of Patients for modelling admissions in emergency (n = 228)

Characteristic	*n(%)*	*Admitted n(%)*	*Not Admitted n(%)*
*Admission Status*			
Admitted	117 (51.3%)	-	-
Not Admitted	111 (48.7%)	-	-
***Age (mean (SD/SE))***	30.0 (14.7)	33.7 (1.7)	26.2 (0.8)
***Year***			
2011	9 (3.9%)	4 (3.4%)	5 (4.5%)
2012	36 (15.8%)	12 (10.3%)	24 (21.6%)
2013	12 (5.3%)	6 (5.1%)	6 (5.4%)
2014	16 (7.0%)	10 (8.6%)	6 (5.4%)
2015	17 (7.5%)	10 (8.6%)	7 (6.3%)
2016	12 (5.3%)	8 (6.8%)	4 (3.6%)
2017	20 (8.8%)	14 (12.0%)	6 (5.4%)
2018	30 (13.2%)	21 (18.0%)	9 (8.1%)
2019	48 (21.1%)	22 (18.8%)	26 (23.4%)
2020	28 (12.3%)	10 (8.6%)	18 (16.2%)
***Eating Disorder Diagnoses***			
Anorexia Nervosa	98 (43.0%)	56 (47.9%)	42 (37.8%)
Bulimia Nervosa	23 (10.1%)	7 (6.0%)	16 (14.4%)
Other	107 (46.9%)	54 (46.2%)	53 (47.8%)
***Facility***			
RPA	193 (84.6%)	98 (83.8%)	95 (85.6%)
Not RPA	35 (15.4%)	19 (16.2%)	16 (14.4%)
***Triage Category***			
Emergency	37 (16.2%)	25 (21.4%)	12 (10.8%)
Urgent	144 (63.2%)	77 (65.8%)	67 (60.4%)
Semi-urgent	44 (19.3%)	15 (21.8%)	29 (26.1%)
Non-urgent	3 (1.3%)	0 (0.0%)	3 (2.7%)
***Referral Source***			
Self/Family/Friends	160 (70.2%)	78 (66.7%)	82 (73.9%)
Other	68 (29.8%)	39 (33.3%)	29 (26.1%)
***Mode of Arrival***			
Ambulance	59 (25.9%)	43 (36.8%)	16 (14.4%)
Not Ambulance	169 (74.1%)	74 (63.3%)	95 (85.6%)
***Gender***			
Male	18 (7.9%)	11 (9.4%)	7 (6.3%)
Female	210 (92.1%)	106 (90.6%)	104 (93.7%)
***Marital Status***			
Married/De facto	190 (83.3%)	93 (79.5%)	97 (87.4%)
Not Married/De facto	38 (16.7%)	24 (20.5%)	14 (12.6%)
***Index of Relative Socioeconomic Disadvantage***			
Quintile 1 – Least disadvantaged	28 (12.3%)	14 (12.0%)	14 (12.6%)
Quintile 2	13 (5.7%)	4 (3.4%)	9 (8.1%)
Quintile 3	41 (18.0%)	19 (16.2%)	22 (19.8%)
Quintile 4	52 (22.8%)	27 (23.1%)	25 (22.5%)
Quintile 5 – Most disadvantaged	94 (41.2%)	53 (45.3%)	41 (37.0%)

### Initial Exploratory Model (Modelling Admissions in Emergency)

The multivariable model (Table 2) shows weak evidence of a difference in odds of admission across year (p = 0.089). Odds of admission in 2020 ([OR] 0.18, 95% Confidence Interval [95%CI] 0.05 to 0.64; p = 0.008) and 2012 ([OR] 0.26, 95% Confidence Interval [95% CI] 0.08 to 0.85; p = 0.026) are lower compared to 2018. This year was chosen as the reference year as data collection and policy changes were implemented in 2016, improving consistency in the years 2017 to 2019, so the median from these years was chosen. This is consistent with descriptive statistics (Table 1) and univariate analyses (Supplementary 2). There is also weak evidence of a difference in odds of admission for varying diagnosis, and the odds of admission for patients diagnosed with Bulimia Nervosa is lower ([OR] 0.31, 95% Confidence Interval [95% CI] 0.10 to 0.95; p = 0.040) compared to Anorexia Nervosa. For every unit increase in age there is increase in odds of admission ([OR] 1.04, 95% Confidence Interval [95% CI] 1.01 to 1.08; p = 0.015). Across triage categories (increasing urgency) there is decrease in odds of admission ([OR] 0.37, 95% Confidence Interval [95% CI] 0.21 to 0.66; p = 0.001) for each level of category declining in urgency. There is also difference in odds of an admission dependent on source of referral (self/family or other) and if arriving by ambulance as opposed to self-presenting to Emergency.

**Table 2:** Initial exploratory model (multivariable logistic regression) with outcome as odds ratio of admissions

	Initial Exploratory Model					
*Variable*	*Odds Ratio*	*SE*	*Z*	*p*	*95% CI (Lower)*	*95% CI (Upper)*
***Year (Ref: 2018)***	-	-	-	0.089*	-	-
2011	0.49	0.44	-0.80	0.425	0.08	2.87
2012	0.26	0.16	-2.23	0.026	0.08	0.85
2013	0.44	0.35	-1.04	0.299	0.09	2.09
2014	0.89	0.68	-0.15	0.882	0.20	4.01
2015	0.79	0.56	-0.33	0.738	0.20	3.15
2016	1.16	0.96	0.18	0.857	0.23	5.85
2017	0.81	0.58	-0.30	0.767	0.20	3.27
2019	0.34	0.19	-1.93	0.054	0.11	1.02
2020	0.18	0.12	-2.67	0.008	0.05	0.64
***Eating Disorder Diagnoses (Ref: Anorexia Nervosa)***	-	-	-	0.065*	-	-
Bulimia Nervosa	0.31	0.18	-2.05	0.04	0.10	0.95
Other	0.58	0.20	-1.62	0.105	0.30	1.12
***Age***	1.04	0.02	2.43	0.015	1.01	1.08
***Facility (Ref: Not RPA)***						
RPA	0.73	0.35	-0.65	0.513	0.28	1.89
***Triage Category***	0.37	0.11	-3.39	0.001	0.21	0.66
***Referral Source (Ref: Self/Family/Friends)***						
Other	2.03	0.74	1.96	0.050	1.00	4.13
***Mode of Arrival (Ref: Not Ambulance)***						
Ambulance	2.28	0.88	2.15	0.032	1.07	4.86
***Gender (Ref: Male)***						
Female	0.60	0.42	-0.73	0.465	0.15	2.36
***Marital Status (Ref: Not Married/De facto)***						
Married/De facto	1.01	0.51	0.02	0.984	0.38	2.71
***Index of Relative Socioeconomic Disadvantage***	1.17	0.14	1.34	0.179	0.93	1.48

*Wald Test for significance of multinomial variables

For testing goodness of fit, the Hosmer Lemeshow Test had a p-value of 0.784, indicating the model fits well. When compared to the minimal model there is a significant difference to the multivariable model with p < 0.001, which demonstrates a better fit. These tests are also supported by the area under the receiver operating characteristic curve (AUC) of 0.788 which indicates acceptable discrimination. The pseudo R^2^ is 0.201 which suggests approximately 20.1% proportional improvement compared to the minimal model.

### Final Prediction Model (Modelling Admissions in Emergency)

A final prediction model for predicting ED admissions in emergency department was developed with results of the model in Table 3. This has a pseudo R^2^ of 0.135 which suggests approximately 13.5% proportional improvement compared to the minimal model. For goodness of fit, the Hosmer Lemeshow Test has a p-value of 0.249, which provides evidence the model fits well. Furthermore, this final prediction model is internally validated by bootstrapping with 1000 repetitions. (Supplementary 3) Also, compared to the minimal model there is a significant difference to the multivariable model with p < 0.001. These tests are also supported by the AUC of 0.724 which indicates acceptable discrimination. Although not statistically significant at the 5% level of significance as a predictor (p = 0.09), ED diagnoses are retained as a variable as it is considered a clinically important variable.

**Table 3:** Final prediction model (multivariable logistic regression)

	Final Prediction Model					
*Variables*	*Odds Ratio*	*SE*	*Z*	*p*	*95% CI (Lower)*	*95% CI (Upper)*
***Eating Disorder Diagnoses (Ref: Anorexia Nervosa)***	-	-	-	0.09*	-	-
Bulimia Nervosa	0.35	0.18	-2.02	0.044	0.12	0.97
Other	0.65	0.20	-1.42	0.157	0.35	1.18
***Age***	1.05	0.01	3.38	0.001	1.02	1.07
***Triage Category***	0.45	0.11	-3.17	0.002	0.27	0.73
***Mode of Arrival (Ref: Not Ambulance)***						
Ambulance	2.38	0.85	2.42	0.015	1.18	4.79

*Wald Test for significance of multinomial variables

### Patient Characteristics (Modelling Length of Stay)

There were 613 patients from the APD identified as diagnosed with ED between 2014 and 2020. The mean age is 30.7 years (SD = 13.4, range 16 to 86). Peak number of admissions were in 2019 with over two thirds of presentations identified with a diagnosis of AN. Most patients were recorded as female (93.3%) and presented to RPA (81.2%). A number of diagnostic categories were identified from grouping ICD-10-AM codes and whilst some had low prevalence, all clinically important and relevant diagnostic categories were retained for analysis. The complete characteristics are in Table 4.

**Table 4:** Characteristics of Patients for modelling length of stay (n = 613)

Characteristic	*n(%)*	Diagnostic Categories	*n(%)*
***Length of Stay (median (IQR))***	*17 (5–50)*	***Mood Disorder***	
***Year***		Yes	246 (40.1%)
2014	24 (3.9%)	No	367 (59.9%)
2015	84 (13.7%)	***Psychotic Disorder***	
2016	79 (12.9%)	Yes	33 (5.4%)
2017	89 (14.5%)	No	580 (94.6%)
2018	107 (17.5%)	***Substance Disorder***	
2019	131 (21.4%)	Yes	168 (27.4%)
2020	99 (16.2%)	No	445 (72.6%)
***Diagnosis Counts (Median (IQR))***	7 (5–11)	***Personality Disorders***	
***Age (Mean (SD))***	30.7 (13.4)	Yes	146 (23.8%)
***Eating Disorder Diagnoses***		No	467 (76.2%)
Anorexia Nervosa	413 (67.4%)	***Anxiety Disorders***	
Bulimia Nervosa	76 (12.4%)	Yes	154 (25.1%)
Other	124 (20.2%)	No	459 (74.9%)
***Eating Disorder Diagnosis Type***		***Self-Harm/Suicide***	
Principle	318 (51.9%)	Yes	117 (19.1%)
Not Principle	295 (48.1%)	No	496 (80.9%)
***Medical Ward as part of admission***		***Adjustment Disorder***	
Yes	251 (41.0%)	Yes	55 (9.0%)
No	362 (59.0%)	No	558 (91.0%)
***Mental Health Ward as part of admission***		***Childhood Psychiatric Disorders***	
Yes	208 (33.9%)	Yes	9 (1.5%)
No	405 (66.1%)	No	604 (98.5%)
***Specialist ED Ward***		***Behavioural Disorders***	
Yes	238 (38.8%)	Yes	50 (8.2%)
No	375 (61.2%)	No	563 (91.8%)
**Facility**		***Delirium/Dementias***	
RPA	498 (81.2%)	Yes	11 (1.8%)
Not RPA	115 (18.8%)	No	602 (98.2%)
***Mode of Separation***		***Phosphate/Oedema***	
Discharged by hospital	480 (78.3%)	Yes	63 (10.3%)
Discharged at own risk	35 (5.7%)	No	550 (89.7%)
Transfer Outside Service	53 (8.7%)	***Acute Malnutrition Markers***	
Transfer Within Service	45 (7.3%)	Yes	406 (66.2%)
***Treatment in Emergency***		No	207 (33.8%)
Yes	257 (41.9%)	***Effects of Chronic Malnutrition***	
No	356 (58.1%)	Yes	117 (19.1%)
***Referral Source***		No	496 (80.9%)
Emergency	252 (41.1%)	***Gastrointestinal Issues***	
Not Emergency	361 (58.9%)	Yes	188 (30.7%)
***Intensive Care Unit as part of admission***		No	425 (69.3%)
Yes	38 (6.2%)	***Hypokalemia/Alkalosis***	
No	575 (93.8%)	Yes	88 (14.4%)
***Gender***		No	525 (85.6%)
Male	41 (6.7%)	***Dehydration***	
Female	572 (93.3%)	Yes	95 (15.5%)
***Marital Status***		No	518 (84.5%)
Married/De facto	73 (11.9%)	***Other Electrolyte Disturbances***	
Not Married/De facto	540 (88.1%)	Yes	50 (8.2%)
***Index of Relative Socioeconomic Disadvantage***		No	563 (91.8%)
Quintile 1 – Least disadvantaged	53 (8.7%)	***Cardiac Sequelae***	
Quintile 2	39 (6.4%)	Yes	119 (19.4%)
Quintile 3	143 (23.3%)	No	494 (80.6%)
Quintile 4	176 (28.7%)	***Low Blood Pressure***	
Quintile 5 – Most disadvantaged	202 (33.0%)	Yes	169 (27.6%)
		No	444 (72.4%)
		***Hypoglycemia***	
		Yes	104 (17.0%)
		No	509 (83.0%)

### Initial Exploratory Model (Modelling Length of Stay)

There is strong evidence of a difference in average length of stay across admission years in the initial exploratory model (p = 0.003). Compared to 2018, there is evidence of an average increase of 14.29 days length of stay in 2014 (95% Confidence Interval [95% CI] 2.78 to 25.80; p = 0.015). Also, the average length of stay increased by 0.96 (95% Confidence Interval [95% CI] 0.37 to 1.55; p = 0.001) for every unit increase in diagnosis count. Increased length of stay is also associated with admissions that included a mental health ward or Specialist ED Ward. There is also evidence of a difference in length of stay dependent on the ED diagnoses (p = 0.023) and mode of separation (p < 0.001).

There is an increase in average length of stay if a patient has delirium/dementias 21.83 days (95% Confidence Interval [95% CI] 5.81 to 37.86; p = 0.008), effects of chronic malnutrition 13.77 days (95% Confidence Interval [95% CI] 8.03 to 19.50; p < 0.001), refeeding syndrome markers (hypophosphatemia/oedema) 10.93 days (95% Confidence Interval [95% CI] 3.90 to 17.96; p = 0.002), hypoglycemia 6.77 days (95% Confidence Interval [95% CI] 1.00 to 12.53; p = 0.021), and anxiety disorders 5.68 days (95% Confidence Interval [95% CI] 0.10 to 11.26; p = 0.046). In contrast there is decreased average length of stay if a patient has adjustment disorders − 11.6 days (95% Confidence Interval [95% CI] -20.06 to -3.13; p = 0.007), substance disorders − 6.66 days (95% Confidence Interval [95% CI] -11.45 to -1.88; p = 0.006), and personality disorders − 6.02 (95% Confidence Interval [95% CI] -11.45 to -0.58; p = 0.030).

The R^2^ for this initial exploratory model (Table 5) is 0.587, which indicates that 58.7% of variability in length of stay is explained by the variables in this model. It is observed from the validation tests that there is heteroscedasticity and some minor departure from normality of errors. However, with a large sample (n > 500), it has been shown that even the most skewed data can be analysed using least squares regression [27]. Sensitivity analyses were also performed comparing this model to negative binomial models. (Supplementary 5) Despite some differences in output, the majority of the output were similar, further supporting the linear model.

**Table 5:** Initial exploratory model (multivariable linear regression) with outcome as length of stay

	Initial Exploratory Model					
*Variables*	*Coefficient*	*SE*	*t*	*p*	*95% CI (Lower)*	*95% CI (Upper)*
***Year (Ref: 2018)***	-	-	-	0.003*	-	-
2014	14.29	5.86	2.44	0.015	2.78	25.80
2015	-3.14	3.75	-0.84	0.403	-10.49	4.22
2016	7.23	3.80	1.90	0.058	-0.24	14.69
2017	-0.07	3.57	-0.02	0.983	-7.10	6.95
2019	-1.76	3.30	-0.53	0.595	-8.23	4.72
2020	-5.21	3.57	-1.46	0.145	-12.22	1.80
***Diagnosis Counts***	0.96	0.30	3.21	0.001	0.37	1.55
***Age (Centred)***	-0.10	0.09	-1.18	0.237	-0.28	0.07
***Eating Disorder Diagnoses (Ref: Anorexia Nervosa)***	-	-	-	0.023*	-	-
Bulimia Nervosa	-9.05	3.34	-2.71	0.007	-15.60	-2.49
Other	-3.46	2.93	-1.18	0.239	-9.22	2.30
***Eating Disorder Diagnosis Type (Ref: Not Principle)***						
Principle	-1.68	3.09	-0.54	0.587	-7.74	4.38
***Medical Ward (Ref: No Medical Ward)***						
Medical Ward	-1.29	4.18	-0.31	0.758	-9.49	6.92
***Mental Health Ward (Ref: No Mental Health Ward)***						
Mental Health Ward	9.03	4.06	2.23	0.026	1.06	17.00
***Specialist ED Ward (Ref: No Specialist ED Ward)***						
Specialist ED Ward	47.05	4.70	10.00	< 0.001	37.81	56.29
***Facility (Ref: Not RPA)***						
RPA	-1.27	3.02	-0.42	0.673	-7.20	4.66
***Mode of Separation (Ref: Discharged by hospital)***	-	-	-	< 0.001*	-	-
Discharged at own risk	-14.92	4.42	-3.38	0.001	-23.59	-6.24
Transfer Outside Service	-3.56	3.70	-0.96	0.336	-10.82	3.70
Transfer Within Service	22.43	5.18	4.33	< 0.001	12.26	32.61
***Referral Source (Ref: Emergency)***						
Not Emergency	0.35	2.81	0.13	0.899	-5.16	5.86
***Intensive Care Unit (Ref: Not ICU)***						
ICU	-0.79	4.56	-0.17	0.863	-9.74	8.16
***Gender (Ref: Male)***						
Female	-5.62	4.16	-1.35	0.178	-13.79	2.56
***Marital Status (Ref: Not Married/De facto)***						
Married/De facto	-6.07	3.23	-1.88	0.061	-12.42	0.27
***Index of Relative Socioeconomic Disadvantage***	1.51	0.86	1.76	0.079	-0.17	3.19
***Diagnosis Categories (Ref: No)***						
***Mood Disorder***	2.88	2.20	1.31	0.191	-1.44	7.21
***Psychotic Disorder***	7.02	4.74	1.48	0.140	-2.30	16.33
***Substance Disorder***	-6.66	2.44	-2.73	0.006	-11.45	-1.88
***Personality Disorders***	-6.02	2.77	-2.17	0.030	-11.45	-0.58
***Anxiety Disorders***	5.68	2.84	2.00	0.046	0.10	11.26
***Self-Harm/Suicide***	-2.20	3.22	-0.68	0.496	-8.53	4.14
***Adjustment Disorder***	-11.60	4.31	-2.69	0.007	-20.06	-3.13
***Childhood Psychiatric Disorders***	10.28	8.39	1.22	0.221	-6.20	26.76
***Behavioural Disorders***	0.19	3.89	0.05	0.960	-7.44	7.83
***Delirium/Dementias***	21.83	8.16	2.68	0.008	5.81	37.86
***Phosphate/Oedema***	10.93	3.58	3.05	0.002	3.90	17.96
***Acute Malnutrition Markers***	-2.18	2.82	-0.77	0.439	-7.71	3.35
***Effects of Chronic Malnutrition***	13.77	2.92	4.71	< 0.001	8.03	19.50
***Gastrointestinal Issues***	2.28	2.39	0.95	0.341	-2.41	6.97
***Hypokalemia/Alkalosis***	-2.14	3.23	-0.66	0.507	-8.48	4.20
***Dehydration***	-1.67	3.10	-0.54	0.591	-7.76	4.43
***Cardiac Sequelae***	4.28	2.80	1.53	0.128	-1.23	9.79
***Low Blood Pressure***	4.30	2.65	1.62	0.105	-0.91	9.51
***Hypoglycemia***	6.77	2.93	2.31	0.021	1.00	12.53

*Wald Test for significance of multinomial variables

### Final Prediction Model (Modelling Length of Stay)

A final prediction model for length of stay for patients admitted with ED was developed with results of the model in Table *6*. The R^2^ for this final prediction model is 0.562, which indicates that 56.2% of variability in length of stay is explained by inclusion of all these variables in this final prediction model. This final prediction model is internally validated by bootstrapping. (Supplementary 6) Furthermore, the same validation process and sensitivity analyses were conducted as the initial exploratory model with similar results providing support for a linear model. (Supplementary 7)

## Discussion

To assess the utility of routinely collected health administrative data to model associations and predict admissions and length of stay of patients being treated for ED, investigators built two models from each data set. A full multivariable model to explore associations on patients admitted from emergency (Table 2) and length of stay of admitted patients (Table 4) from the SLHD Emergency Department Data (EDD) and SLHD Admitted Patient Data (APD) respectively. Following this, a main effects model for prediction was developed (Tables 3 and 6) by process of “Purposeful Selection of Covariates”. The results were mixed, consistent with several other papers [28, 29]. There is some utility in modelling admissions in emergency with factors associated with admissions in the initial exploratory model. However, there is more utility in modelling length of stay both on associations with the initial exploratory model as well as the final prediction model.

**Table 6:** Final prediction model (multivariable linear regression)

	Final Prediction Model					
*Variables*	*Coefficient*	*SE*	*t*	*p*	*95% CI (Lower)*	*95% CI (Upper)*
***Diagnosis Counts***	1.17	0.21	5.48	< 0.001	0.75	1.59
***Eating Disorder Diagnoses (Ref: Anorexia Nervosa)***	-	-	-	0.032*	-	-
Bulimia Nervosa	-8.51	3.33	-2.56	0.011	-15.05	-1.98
Other	-3.68	2.88	-1.28	0.202	-9.33	1.97
***Medical Ward (Ref: No Medical Ward)***						
Medical Ward	9.67	3.22	3.01	0.003	3.35	15.98
***Mental Health Ward (Ref: No Mental Health Ward)***						
Mental Health Ward	19.42	3.53	5.50	< 0.001	12.48	26.36
***Specialist ED Ward (Ref: No Specialist ED Ward)***						
Specialist ED Ward	56.45	3.36	16.81	< 0.001	49.85	63.04
***Marital Status (Ref: Not Married/De facto)***						
Married/De facto	-7.51	3.21	-2.34	0.020	-13.82	-1.19
***Index of Relative Socioeconomic Disadvantage***	1.82	0.85	2.15	0.032	0.16	3.49
***Substance Disorder***	-8.12	2.39	-3.39	0.001	-12.82	-3.42
***Personality Disorders***	-7.09	2.66	-2.66	0.008	-12.32	-1.85
***Anxiety Disorders***	7.57	2.87	2.64	0.008	1.94	13.20
***Adjustment Disorder***	-11.64	4.37	-2.66	0.008	-20.22	-3.05
***Phosphate/Oedema***	11.30	3.55	3.18	0.002	4.33	18.26
***Effects of Chronic Malnutrition***	11.23	2.88	3.90	< 0.001	5.58	16.88

This is the first of further analyses and models that will be developed to provide health services with a potentially innovative and cost-effective way to better understand risk factors and predict health care outcomes of people with ED. In turn, this could lead to standardisation of data collection and improve the utility of routinely collected health administrative data. This can provide valuable information to a more predictive approach to healthcare, effective and efficient allocation of resources and guide improvements in service delivery for people with ED, their families, and carers. It is expected that these models will be the first steps in exploring routinely collected health administrative data in eating disorders and evaluate its clinical utility by developing, evaluating, and validating exploratory and prediction models.

### Modelling Admissions in Emergency

In this study modelling emergency department admissions utilising EDD, the initial exploratory model consists of 10 variables and after purposeful selection of covariates, the final prediction model consists of 4 variables. In other studies on prediction models on emergency department admissions, variables included in these models varied significantly such as diagnoses, functional status and quality of life [28, 30]. The source of data utilised also varied, including administrative, clinical and survey data [31–33]. It has been found that administrative or clinical data have greater predictive ability compared to survey data [28].

In the initial exploratory model (Table 2), ED diagnoses, age, triage category, source of referral and mode of arrival are observed to be statistically significant factors impacting on admission. For example, with every year increase in age there is 4% greater odds of admission (95% Confidence Interval [95% CI] 1.01 to 1.08; p = 0.015). Whilst there are limitations with the validation by Hosmer Lemeshow test, results from the model are clinically plausible and valid both in magnitude and direction. Investigators believe this contributes to the validity of the model and provides evidence administrative data can be utilised to understand factors that impact on ED patient admissions in emergency.

However, the same cannot be inferred from the final prediction model (Table 3) as it consists of only 4 variables, ED diagnoses, age, triage category and mode of arrival. Whilst each variable is clinically valid, they would not be considered adequate to predict admissions, supported by the pseudo R^2^ of 0.135, which is relatively low. Several other studies have shown multiple comorbidities and polypharmacy have a significant impact of health care utilisation [34, 35], which were not available in the EDD and may have improved the utility/validity of the final prediction model.

### Modelling Length of Stay

The initial exploratory model for length of stay has clinically plausible and valid factors that impact on the outcome of length of stay. This initial exploratory model includes 34 variables in total in which 19 are diagnostic categorisations based on ICD-10-AM. It is clear that the APD contains much further information than EDD, including the important variable of diagnoses, which is not available in the EDD.

Diagnoses are important variables in health care outcomes and utilisation. A large number of studies on length of stay have utilised Charlson Comorbidity Index and other methods of indexing diagnoses with mixed results [31]. There are numerous adaptations of Charlson Comorbidity Index [36–38] however none that exist for ED and given clinical heterogeneity in this population, utilising such an index would require adaptation and validation. Therefore, diagnoses were included in a number of ways including diagnosis counts, eating disorder diagnoses and clinically appropriate and meaningful groupings and categorisations of comorbid diagnoses. Furthermore, from the initial exploratory model in the results Table 5, there is further evidence of clinical utility as a number of clinically plausible variables are associated with length of stay in addition to diagnoses related variables including admission year, type of wards admitted and mode of separation.

In the final prediction model in Table 6, diagnoses of substance disorder, personality disorder, anxiety disorder, adjustment disorder, refeeding syndrome markers (hypophosphatemia/oedema) and effects of chronic malnutrition provide predictive value. As well as the diagnoses, predictors for length of stay include diagnosis counts, ED diagnoses, type of wards admitted, marital status and socioeconomic disadvantage. The variables in the final prediction model for length of stay are all considered to be clinically plausible and valid and this is again supported statistically by the model with R^2^ of 0.562 as well as internal validation via bootstrapping (Supplementary 6). Based on the R^2^, this model performed relatively well compared to numerous other studies on prediction models for length of stay, which had R^2^ between 0.3 and 0.6 [29]. Many studies are now utilising machine learning models used for predicting length of stay, however the model in this study performed better than most of the machine learning models thus far [39].

### Limitations

The purpose of this study is to assess the clinical utility of health administrative data but there are several limitations in using these data. These data are not purposefully collected for this study or for clinical purposes. It is collected for resource utilisation and allocations with data entry initially, by administrative staff and clinicians with limited training on data entry. There is a lack of standardisation in data collection in EDD in contrast to APD, where data collection is conducted by highly trained coders reviewing all the documentation. This, in part, explains the improved validity and utility of modelling length of stay compared to admissions in emergency.

For modelling admissions in emergency, the sample size was much smaller than anticipated as the search criteria was based on SNOMED diagnosis codes. It is known that patients with ED present to emergency department for numerous physical or mental health issues such as electrolyte disturbance or self-harm, which would not have been identified and this is a known limitation of SNOMED codes [40]. Therefore, the population was defined as patients with confirmed ED diagnoses on presentation to emergency for this study to better understand limitations of EDD data with the view to inform follow up studies, further analyses and models.

From modelling length of stay, the diagnostic groupings were based on ICD-10-AM coding, which is not consistent with diagnostic criteria utilised in clinical practice and did not include severity. This could potentially impact predictive value for length of stay. However, some limitations were mitigated by meticulously reviewing each of the ICD-10-AM codes with these diagnostic groupings both statistically and clinically, with multidisciplinary clinical input in a cyclical iterative process.

### Conclusions and Future Directions

Health administrative data can be utilised to describe factors that impact on health outcome measures as well as predict health outcome measures in adults with ED. Both the initial exploratory models provide useful and clinically valid information. The final prediction model for admissions in emergency is not statistically or clinically valid for prediction, but there is potential with inclusion of further variables such as diagnoses. This is demonstrated by the final prediction model for length of stay, which performed well in comparison to other studies on prediction models for length of stay. Further exploration and evaluation of this type of data with inclusion of more information from data linkage and clinical information evaluating various statistical methods are the next steps currently in progress following this study. Furthermore, future studies will also include external validation, translation and implementation with prospective studies to further validate and optimise the final prediction models.

It is also evident from this study that there would be numerous benefits to inclusion of further variables and standardising data collection. Utilising health administrative data in this way is an efficient process for assessing impacts of multiple factors on patient care and predicting their health care outcomes, which has significant potential in not only guiding effective resource allocation and utilisation but also improving care provided to people with a lived experience of ED, their families and carers. It is even more important now to use all available data to optimise outcomes and provide efficacious and cost-effective services to meet the growing demand for treatment and care in the community, especially given the ongoing increasing prevalence and impacts of COVID-19 in mental health and eating disorders.

## Electronic supplementary material

Below is the link to the electronic supplementary material.

Supplementary Material 1

## Data Availability

The datasets generated and/or analysed during the current study are not publicly available due to privacy and ethical restrictions as per NSW Health Policy but may be available from the corresponding author on reasonable request.

## Abbreviations

DSM-5: Diagnostic and Statistical Manual of Mental Disorders (version 5).
AN: Anorexia Nervosa.
BN: Bulimia Nervosa.
ARFID: Avoidant/Restrictive Food Intake Disorder.
USFED: Unspecified Feeding or eating disorder.
OSFED: Other Specified Feeding or eating disorder.
SLHD: Sydney Local Health District.
EDDC: NSW Emergency Department Data Collection.
APDC: NSW Admitted Patient Data Collection.
EDD: SLHD Emergency Department Data.
APD: SLHD Admitted Patient Data.
ICD-10-AM: International Classification of Diseases, Tenth Revision (Australian Modification).
SEIFA: Socio-Economic Indexes for Australia, 2016.
