# Supplementary material for: Using health administrative data to model associations and predict hospital admissions and length of stay for people with eating disorders
Source: BMC Psychiatry. 2023 May 10;23:326. doi: 10.1186/s12888-023-04688-x (PMC10170048; doi:10.1186/s12888-023-04688-x)
Supplement: Supplementary file 1 — Supplementary Material 1 [file 12888_2023_4688_MOESM1_ESM.docx]

**Using health administrative data to model associations and predict hospital admissions and length of stay for people with eating disorders**

**Supplementary 1.** Diagnosis Groupings for APD

| **Diagnosis Grouping Name** | **ICD-10-AM Codes** |
| --- | --- |
| Mood Disorder | U79.3, F30 - 39 and F06.3 |
| Psychotic Disorder | F20 - 29, R44 and U79.2 |
| Substance Disorder | F10 - 19, Z72.0, Z72.1 and Z72.2 |
| Personality Disorders | F60 and F61 |
| Anxiety Disorders | F40 - 48 |
| Self Harm/Suicide | X70 - 84, R45.81, R45.88, S50 - 59 and S60 - 69 |
| Adjustment Disorder | F43 |
| Childhood Psychiatric Disorders | F70 - 79, F80 - 89 and F90 - 98 |
| Behavioural Disorders | F63 - 69, R45 and R46 |
| Delirium/Dementias | F01 - 09 |
| Phosphate/Oedema | E83.3 and R60 |
| Acute Malnutrition Markers | D50 - 53, D69.5, D69.6, D64.9, D70, D72.8, E16.1, E16.2, E40 - 46, E50 - 64, L89, N91, M62.5, R53, R63, R64 and R68.0 |
| Effects of Chronic Malnutrition | M81, M82, M83, M84, M85, U86.4 and E30 |
| Gastrointestinal Issues | K02, K12, K20, K21, K56.41, K59.0, K59.1, K59.8, K64, K76, R11, R12, R15, R19.4, R74 and R94 |
| Hypokalemia/Alkalosis | E87.6 and E87.3 |
| Dehydration | E86, E87.0, N17, E87.2 and E87.5 |
| Cardiac Sequelae | R00, R07, I49 and R94.3 |
| Low Blood Pressure | R55, R42 and I95 |
| Hypoglycemia | E16.1 and E16.2 |

**Supplementary 2.** Univariate analyses and initial exploratory model (multivariable logistic regression) with outcome as odds ratio of admissions

|  | **Univariate Analysis** | | | | | | **Initial Exploratory Model** | | | | | |
| --- | --- | --- | --- | --- | --- | --- | --- | --- | --- | --- | --- | --- |
| ***Variable*** | ***Odds Ratio*** | ***SE*** | ***Z*** | ***p*** | ***95% CI (Lower)*** | ***95% CI (Upper)*** | ***Odds Ratio*** | ***SE*** | ***Z*** | ***p*** | ***95% CI (Lower)*** | ***95% CI (Upper)*** |
| ***Year (Ref: 2018)*** | - | - | - | 0.053* | - | - | - | - | - | 0.089* | - | - |
| 2011 | 0.34 | 0.27 | -1.37 | 0.170 | 0.07 | 1.58 | 0.49 | 0.44 | -0.80 | 0.425 | 0.08 | 2.87 |
| 2012 | 0.21 | 0.11 | -2.89 | 0.004 | 0.08 | 0.61 | 0.26 | 0.16 | -2.23 | 0.026 | 0.08 | 0.85 |
| 2013 | 0.43 | 0.30 | -1.21 | 0.227 | 0.11 | 1.69 | 0.44 | 0.35 | -1.04 | 0.299 | 0.09 | 2.09 |
| 2014 | 0.71 | 0.47 | -0.52 | 0.606 | 0.20 | 2.56 | 0.89 | 0.68 | -0.15 | 0.882 | 0.20 | 4.01 |
| 2015 | 0.61 | 0.39 | -0.77 | 0.439 | 0.18 | 2.12 | 0.79 | 0.56 | -0.33 | 0.738 | 0.20 | 3.15 |
| 2016 | 0.86 | 0.63 | -0.21 | 0.833 | 0.20 | 3.59 | 1.16 | 0.96 | 0.18 | 0.857 | 0.23 | 5.85 |
| 2017 | 1.00 | 0.63 | 0.00 | 1.000 | 0.29 | 3.44 | 0.81 | 0.58 | -0.30 | 0.767 | 0.20 | 3.27 |
| 2019 | 0.36 | 0.18 | -2.06 | 0.039 | 0.14 | 0.95 | 0.34 | 0.19 | -1.93 | 0.054 | 0.11 | 1.02 |
| 2020 | 0.24 | 0.13 | -2.56 | 0.010 | 0.08 | 0.71 | 0.18 | 0.12 | -2.67 | 0.008 | 0.05 | 0.64 |
| ***Eating Disorder Diagnoses (Ref: Anorexia Nervosa)*** | - | - | - | 0.077* | - | - | - | - | - | 0.065* | - | - |
| Bulimia Nervosa | 0.33 | 0.16 | -2.24 | 0.025 | 0.12 | 0.87 | 0.31 | 0.18 | -2.05 | 0.04 | 0.10 | 0.95 |
| Other | 0.76 | 0.21 | -0.96 | 0.339 | 0.44 | 1.33 | 0.58 | 0.20 | -1.62 | 0.105 | 0.30 | 1.12 |
| ***Age*** | 1.04 | 0.01 | 3.54 | <0.001 | 1.02 | 1.07 | 1.04 | 0.02 | 2.43 | 0.015 | 1.01 | 1.08 |
| ***Facility (Ref: Not RPA)*** |  |  |  |  |  |  |  |  |  |  |  |  |
| RPA | 0.87 | 0.32 | -0.38 | 0.703 | 0.42 | 1.79 | 0.73 | 0.35 | -0.65 | 0.513 | 0.28 | 1.89 |
| ***Triage Category*** | 0.46 | 0.11 | -3.37 | 0.001 | 0.30 | 0.73 | 0.37 | 0.11 | -3.39 | 0.001 | 0.21 | 0.66 |
| ***Referral Source (Ref: Self/Family/Friends)*** |  |  |  |  |  |  |  |  |  |  |  |  |
| Other | 1.41 | 0.41 | 1.19 | 0.235 | 0.80 | 2.50 | 2.03 | 0.74 | 1.96 | 0.050 | 1.00 | 4.13 |
| ***Mode of Arrival (Ref: Not Ambulance)*** |  |  |  |  |  |  |  |  |  |  |  |  |
| Ambulance | 3.45 | 1.14 | 3.74 | <0.001 | 1.80 | 6.61 | 2.28 | 0.88 | 2.15 | 0.032 | 1.07 | 4.86 |
| ***Gender (Ref: Male)*** |  |  |  |  |  |  |  |  |  |  |  |  |
| Female | 0.65 | 0.33 | -0.86 | 0.389 | 0.24 | 1.74 | 0.60 | 0.42 | -0.73 | 0.465 | 0.15 | 2.36 |
| ***Marital Status (Ref: Not Married/De facto)*** |  |  |  |  |  |  |  |  |  |  |  |  |
| Married/De facto | 0.56 | 0.20 | -1.59 | 0.113 | 0.27 | 1.15 | 1.01 | 0.51 | 0.02 | 0.984 | 0.38 | 2.71 |
| ***Index of Relative Socioeconomic Disadvantage*** | 1.13 | 0.11 | 1.28 | 0.200 | 0.94 | 1.37 | 1.17 | 0.14 | 1.34 | 0.179 | 0.93 | 1.48 |

*Wald Test for significance of multinomial variables

**Supplementary 3**. Final prediction model (multivariable logistic regression) and bootstrap internal validation with 1000 bootstrap repetitions

|  | **Final Prediction Model** | | | | | | **Bootstrapped Estimates** | | | | |
| --- | --- | --- | --- | --- | --- | --- | --- | --- | --- | --- | --- |
| ***Variables*** | ***Odds Ratio*** | ***SE*** | ***Z*** | ***p*** | ***95% CI (Lower)*** | ***95% CI (Upper)*** | ***SE*** | ***Z*** | ***p*** | ***95% CI (Lower)*** | ***95% CI (Upper)*** |
| ***Eating Disorder Diagnoses (Ref: Anorexia Nervosa)*** | - | - | - | 0.09* | - | - | - | - | 0.148* | - | - |
| Bulimia Nervosa | 0.35 | 0.18 | -2.02 | 0.044 | 0.12 | 0.97 | 0.22 | -1.71 | 0.088 | 0.10 | 1.17 |
| Other | 0.65 | 0.20 | -1.42 | 0.157 | 0.35 | 1.18 | 0.21 | -1.35 | 0.177 | 0.34 | 1.22 |
| ***Age*** | 1.05 | 0.01 | 3.38 | 0.001 | 1.02 | 1.07 | 0.01 | 3.45 | 0.001 | 1.02 | 1.07 |
| ***Triage Category*** | 0.45 | 0.11 | -3.17 | 0.002 | 0.27 | 0.73 | 0.12 | -2.94 | 0.003 | 0.26 | 0.76 |
| ***Mode of Arrival (Ref: Not Ambulance)*** |  |  |  |  |  |  |  |  |  |  |  |
| Ambulance | 2.38 | 0.85 | 2.42 | 0.015 | 1.18 | 4.79 | 0.92 | 2.25 | 0.025 | 1.12 | 5.06 |

*Wald Test for significance of multinomial variables

**Supplementary 4.** Univariate analyses and initial exploratory model (multivariable linear regression) with outcome as length of stay

|  | **Univariate Analysis** | | | | | | **Initial Exploratory Model** | | | | | |
| --- | --- | --- | --- | --- | --- | --- | --- | --- | --- | --- | --- | --- |
| ***Variables*** | ***Coefficient*** | ***SE*** | ***t*** | ***p*** | ***95% CI (Lower)*** | ***95% CI (Upper)*** | ***Coefficient*** | ***SE*** | ***t*** | ***p*** | ***95% CI (Lower)*** | ***95% CI (Upper)*** |
| ***Year (Ref: 2018)*** | - | - | - | 0.494* | - | - | - | - | - | 0.003* | - | - |
| 2014 | 3.23 | 8.50 | 0.38 | 0.704 | -13.46 | 19.91 | 14.29 | 5.86 | 2.44 | 0.015 | 2.78 | 25.80 |
| 2015 | -3.00 | 5.48 | -0.55 | 0.585 | -13.77 | 7.77 | -3.14 | 3.75 | -0.84 | 0.403 | -10.49 | 4.22 |
| 2016 | 7.30 | 5.58 | 1.31 | 0.191 | -3.66 | 18.26 | 7.23 | 3.80 | 1.90 | 0.058 | -0.24 | 14.69 |
| 2017 | 6.49 | 5.40 | 1.20 | 0.229 | -4.10 | 17.09 | -0.07 | 3.57 | -0.02 | 0.983 | -7.10 | 6.95 |
| 2019 | -0.82 | 4.90 | -0.17 | 0.867 | -10.45 | 8.81 | -1.76 | 3.30 | -0.53 | 0.595 | -8.23 | 4.72 |
| 2020 | 0.78 | 5.25 | 0.15 | 0.882 | -9.52 | 11.08 | -5.21 | 3.57 | -1.46 | 0.145 | -12.22 | 1.80 |
| ***Diagnosis Counts*** | 1.13 | 0.28 | 4.10 | <0.001 | 0.59 | 1.67 | 0.96 | 0.30 | 3.21 | 0.001 | 0.37 | 1.55 |
| ***Age (Centred)*** | -0.30 | 0.11 | -2.66 | 0.008 | -0.52 | -0.08 | -0.10 | 0.09 | -1.18 | 0.237 | -0.28 | 0.07 |
| ***Eating Disorder Diagnoses (Ref: Anorexia Nervosa)*** | - | - | - | <0.001* | - | - | - | - | - | 0.023* | - | - |
| Bulimia Nervosa | -27.26 | 4.38 | -6.23 | <0.001 | -35.86 | -18.66 | -9.05 | 3.34 | -2.71 | 0.007 | -15.60 | -2.49 |
| Other | -30.29 | 3.59 | -8.43 | <0.001 | -37.34 | -23.23 | -3.46 | 2.93 | -1.18 | 0.239 | -9.22 | 2.30 |
| ***Eating Disorder Diagnosis Type (Ref: Not Principle)*** |  |  |  |  |  |  |  |  |  |  |  |  |
| Principle | 32.12 | 2.75 | 11.68 | <0.001 | 26.72 | 37.52 | -1.68 | 3.09 | -0.54 | 0.587 | -7.74 | 4.38 |
| ***Medical Ward (Ref: No Medical Ward)*** |  |  |  |  |  |  |  |  |  |  |  |  |
| Medical Ward | -13.55 | 3.04 | -4.46 | <0.001 | -19.53 | -7.58 | -1.29 | 4.18 | -0.31 | 0.758 | -9.49 | 6.92 |
| ***Mental Health Ward (Ref: No Mental Health Ward)*** |  |  |  |  |  |  |  |  |  |  |  |  |
| Mental Health Ward | -19.38 | 3.11 | -6.23 | <0.001 | -25.50 | -13.27 | 9.03 | 4.06 | 2.23 | 0.026 | 1.06 | 17.00 |
| ***Specialist ED Ward (Ref: No Specialist ED Ward)*** |  |  |  |  |  |  |  |  |  |  |  |  |
| Specialist ED Ward | 51.49 | 2.32 | 22.19 | <0.001 | 46.93 | 56.05 | 47.05 | 4.70 | 10.00 | <0.001 | 37.81 | 56.29 |
| ***Facility (Ref: Not RPA)*** |  |  |  |  |  |  |  |  |  |  |  |  |
| RPA | 25.30 | 3.76 | 6.74 | <0.001 | 17.92 | 32.67 | -1.27 | 3.02 | -0.42 | 0.673 | -7.20 | 4.66 |
| ***Mode of Separation (Ref: Discharged by hospital)*** | - | - | - | <0.001* | - | - | - | - | - | <0.001* | - | - |
| Discharged at own risk | -12.09 | 6.27 | -1.93 | 0.054 | -24.40 | 0.21 | -14.92 | 4.42 | -3.38 | 0.001 | -23.59 | -6.24 |
| Transfer Outside Service | -10.68 | 5.18 | -2.06 | 0.040 | -20.85 | -0.51 | -3.56 | 3.70 | -0.96 | 0.336 | -10.82 | 3.70 |
| Transfer Within Service | 40.85 | 5.58 | 7.32 | <0.001 | 29.90 | 51.81 | 22.43 | 5.18 | 4.33 | <0.001 | 12.26 | 32.61 |
| ***Referral Source (Ref: Emergency)*** |  |  |  |  |  |  |  |  |  |  |  |  |
| Not Emergency | 24.01 | 2.93 | 8.19 | <0.001 | 18.26 | 29.77 | 0.35 | 2.81 | 0.13 | 0.899 | -5.16 | 5.86 |
| ***Intensive Care Unit (Ref: Not ICU)*** |  |  |  |  |  |  |  |  |  |  |  |  |
| ICU | -0.71 | 6.30 | -0.11 | 0.911 | -13.08 | 11.67 | -0.79 | 4.56 | -0.17 | 0.863 | -9.74 | 8.16 |
| ***Gender (Ref: Male)*** |  |  |  |  |  |  |  |  |  |  |  |  |
| Female | -4.66 | 6.08 | -0.77 | 0.444 | -16.60 | 7.28 | -5.62 | 4.16 | -1.35 | 0.178 | -13.79 | 2.56 |
| ***Marital Status (Ref: Not Married/De facto)*** |  |  |  |  |  |  |  |  |  |  |  |  |
| Married/De facto | -6.11 | 4.69 | -1.30 | 0.193 | -15.31 | 3.09 | -6.07 | 3.23 | -1.88 | 0.061 | -12.42 | 0.27 |
| ***Index of Relative Socioeconomic Disadvantage*** | -2.02 | 1.23 | -1.64 | 0.102 | -4.44 | 0.40 | 1.51 | 0.86 | 1.76 | 0.079 | -0.17 | 3.19 |
| ***Diagnosis Categories (Ref: No)*** |  |  |  |  |  |  |  |  |  |  |  |  |
| ***Mood Disorder*** | -7.79 | 3.08 | -2.53 | 0.012 | -13.85 | -1.74 | 2.88 | 2.20 | 1.31 | 0.191 | -1.44 | 7.21 |
| ***Psychotic Disorder*** | -1.12 | 6.73 | -0.17 | 0.868 | -14.35 | 12.10 | 7.02 | 4.74 | 1.48 | 0.140 | -2.30 | 16.33 |
| ***Substance Disorder*** | -14.44 | 3.36 | -4.30 | <0.001 | -21.03 | -7.85 | -6.66 | 2.44 | -2.73 | 0.006 | -11.45 | -1.88 |
| ***Personality Disorders*** | -11.42 | 3.54 | -3.23 | 0.001 | -18.36 | -4.47 | -6.02 | 2.77 | -2.17 | 0.030 | -11.45 | -0.58 |
| ***Anxiety Disorders*** | 8.41 | 3.49 | 2.41 | 0.016 | 1.56 | 15.26 | 5.68 | 2.84 | 2.00 | 0.046 | 0.10 | 11.26 |
| ***Self-Harm/Suicide*** | -3.81 | 3.86 | -0.99 | 0.324 | -11.40 | 3.78 | -2.20 | 3.22 | -0.68 | 0.496 | -8.53 | 4.14 |
| ***Adjustment Disorder*** | -15.42 | 5.28 | -2.92 | 0.004 | -25.79 | -5.05 | -11.60 | 4.31 | -2.69 | 0.007 | -20.06 | -3.13 |
| ***Childhood Psychiatric Disorders*** | 15.86 | 12.62 | 1.26 | 0.209 | -8.93 | 40.64 | 10.28 | 8.39 | 1.22 | 0.221 | -6.20 | 26.76 |
| ***Behavioural Disorders*** | -10.04 | 5.54 | -1.81 | 0.070 | -20.91 | 0.84 | 0.19 | 3.89 | 0.05 | 0.960 | -7.44 | 7.83 |
| ***Delirium/Dementias*** | 13.14 | 11.44 | 1.15 | 0.251 | -9.32 | 35.60 | 21.83 | 8.16 | 2.68 | 0.008 | 5.81 | 37.86 |
| ***Phosphate/Oedema*** | 22.58 | 4.92 | 4.59 | <0.001 | 12.92 | 32.24 | 10.93 | 3.58 | 3.05 | 0.002 | 3.90 | 17.96 |
| ***Acute Malnutrition Markers*** | 26.24 | 3.03 | 8.65 | <0.001 | 20.29 | 32.20 | -2.18 | 2.82 | -0.77 | 0.439 | -7.71 | 3.35 |
| ***Effects of Chronic Malnutrition*** | 33.40 | 3.62 | 9.22 | <0.001 | 26.28 | 40.52 | 13.77 | 2.92 | 4.71 | <0.001 | 8.03 | 19.50 |
| ***Gastrointestinal Issues*** | 14.43 | 3.24 | 4.45 | <0.001 | 8.06 | 20.80 | 2.28 | 2.39 | 0.95 | 0.341 | -2.41 | 6.97 |
| ***Hypokalemia/Alkalosis*** | -6.25 | 4.33 | -1.44 | 0.149 | -14.75 | 2.24 | -2.14 | 3.23 | -0.66 | 0.507 | -8.48 | 4.20 |
| ***Dehydration*** | -0.70 | 4.20 | -0.17 | 0.868 | -8.94 | 7.55 | -1.67 | 3.10 | -0.54 | 0.591 | -7.76 | 4.43 |
| ***Cardiac Sequelae*** | 13.06 | 3.81 | 3.43 | 0.001 | 5.59 | 20.53 | 4.28 | 2.80 | 1.53 | 0.128 | -1.23 | 9.79 |
| ***Low Blood Pressure*** | 14.08 | 3.35 | 4.20 | <0.001 | 7.49 | 20.66 | 4.30 | 2.65 | 1.62 | 0.105 | -0.91 | 9.51 |
| ***Hypoglycemia*** | 12.94 | 4.02 | 3.22 | 0.001 | 5.06 | 20.83 | 6.77 | 2.93 | 2.31 | 0.021 | 1.00 | 12.53 |

*Wald Test for significance of multinomial variables

**Supplementary 5.** Sensitivity Analysis, Initial exploratory linear model compared to negative binomial model

|  | **Initial Exploratory Linear Model** | | | | | | **Initial Exploratory Negative Binomial Model** | | | | | |
| --- | --- | --- | --- | --- | --- | --- | --- | --- | --- | --- | --- | --- |
| ***Variables*** | ***Coefficient*** | ***SE*** | ***t*** | ***p*** | ***95% CI (Lower)*** | ***95% CI (Upper)*** | ***IRR*** | ***SE*** | ***z*** | ***p*** | ***95% CI (Lower)*** | ***95% CI (Upper)*** |
| ***Year (Ref: 2018)*** | - | - | - | 0.003* | - | - | - | - | - | - | - | - |
| 2014 | 14.29 | 5.86 | 2.44 | 0.015 | 2.78 | 25.80 | 1.84 | 0.49 | 2.29 | 0.022 | 1.09 | 3.09 |
| 2015 | -3.14 | 3.75 | -0.84 | 0.403 | -10.49 | 4.22 | 0.99 | 0.16 | -0.08 | 0.935 | 0.71 | 1.37 |
| 2016 | 7.23 | 3.80 | 1.90 | 0.058 | -0.24 | 14.69 | 1.46 | 0.24 | 2.26 | 0.024 | 1.05 | 2.02 |
| 2017 | -0.07 | 3.57 | -0.02 | 0.983 | -7.10 | 6.95 | 1.11 | 0.17 | 0.68 | 0.497 | 0.82 | 1.51 |
| 2019 | -1.76 | 3.30 | -0.53 | 0.595 | -8.23 | 4.72 | 1.14 | 0.16 | 0.89 | 0.374 | 0.86 | 1.50 |
| 2020 | -5.21 | 3.57 | -1.46 | 0.145 | -12.22 | 1.80 | 1.04 | 0.16 | 0.23 | 0.819 | 0.77 | 1.40 |
| ***Diagnosis Counts*** | 0.96 | 0.30 | 3.21 | 0.001 | 0.37 | 1.55 | 1.05 | 0.01 | 3.19 | 0.001 | 1.02 | 1.07 |
| ***Age (Centred)*** | -0.10 | 0.09 | -1.18 | 0.237 | -0.28 | 0.07 | 1.00 | 0.00 | 0.23 | 0.819 | 0.99 | 1.01 |
| ***Eating Disorder Diagnoses (Ref: Anorexia Nervosa)*** | - | - | - | 0.023* | - | - |  |  |  |  |  |  |
| Bulimia Nervosa | -9.05 | 3.34 | -2.71 | 0.007 | -15.60 | -2.49 | 0.81 | 0.12 | -1.40 | 0.162 | 0.60 | 1.09 |
| Other | -3.46 | 2.93 | -1.18 | 0.239 | -9.22 | 2.30 | 0.86 | 0.11 | -1.14 | 0.253 | 0.67 | 1.11 |
| ***Eating Disorder Diagnosis Type (Ref: Not Principle)*** |  |  |  |  |  |  |  |  |  |  |  |  |
| Principle | -1.68 | 3.09 | -0.54 | 0.587 | -7.74 | 4.38 | 1.03 | 0.14 | 0.23 | 0.815 | 0.79 | 1.35 |
| ***Medical Ward (Ref: No Medical Ward)*** |  |  |  |  |  |  |  |  |  |  |  |  |
| Medical Ward | -1.29 | 4.18 | -0.31 | 0.758 | -9.49 | 6.92 | 0.91 | 0.17 | -0.50 | 0.616 | 0.64 | 1.30 |
| ***Mental Health Ward (Ref: No Mental Health Ward)*** |  |  |  |  |  |  |  |  |  |  |  |  |
| Mental Health Ward | 9.03 | 4.06 | 2.23 | 0.026 | 1.06 | 17.00 | 1.73 | 0.33 | 2.88 | 0.004 | 1.19 | 2.52 |
| ***Specialist ED Ward (Ref: No Specialist ED Ward)*** |  |  |  |  |  |  |  |  |  |  |  |  |
| Specialist ED Ward | 47.05 | 4.70 | 10.00 | <0.001 | 37.81 | 56.29 | 4.50 | 0.91 | 7.41 | <0.001 | 3.03 | 6.71 |
| ***Facility (Ref: Not RPA)*** |  |  |  |  |  |  |  |  |  |  |  |  |
| RPA | -1.27 | 3.02 | -0.42 | 0.673 | -7.20 | 4.66 | 1.08 | 0.15 | 0.55 | 0.583 | 0.83 | 1.41 |
| ***Mode of Separation (Ref: Discharged by hospital)*** | - | - | - | <0.001* | - | - |  |  |  |  |  |  |
| Discharged at own risk | -14.92 | 4.42 | -3.38 | 0.001 | -23.59 | -6.24 | 0.58 | 0.11 | -2.80 | 0.005 | 0.40 | 0.85 |
| Transfer Outside Service | -3.56 | 3.70 | -0.96 | 0.336 | -10.82 | 3.70 | 0.98 | 0.16 | -0.09 | 0.924 | 0.72 | 1.35 |
| Transfer Within Service | 22.43 | 5.18 | 4.33 | <0.001 | 12.26 | 32.61 | 1.61 | 0.37 | 2.11 | 0.035 | 1.03 | 2.51 |
| ***Referral Source (Ref: Emergency)*** |  |  |  |  |  |  |  |  |  |  |  |  |
| Not Emergency | 0.35 | 2.81 | 0.13 | 0.899 | -5.16 | 5.86 | 0.93 | 0.31 | -0.22 | 0.824 | 0.48 | 1.79 |
| ***Intensive Care Unit (Ref: Not ICU)*** |  |  |  |  |  |  |  |  |  |  |  |  |
| ICU | -0.79 | 4.56 | -0.17 | 0.863 | -9.74 | 8.16 | 0.99 | 0.20 | -0.03 | 0.973 | 0.67 | 1.47 |
| ***Gender (Ref: Male)*** |  |  |  |  |  |  |  |  |  |  |  |  |
| Female | -5.62 | 4.16 | -1.35 | 0.178 | -13.79 | 2.56 | 0.89 | 0.16 | -0.69 | 0.491 | 0.63 | 1.25 |
| ***Marital Status (Ref: Not Married/De facto)*** |  |  |  |  |  |  |  |  |  |  |  |  |
| Married/De facto | -6.07 | 3.23 | -1.88 | 0.061 | -12.42 | 0.27 | 0.88 | 0.13 | -0.87 | 0.382 | 0.67 | 1.17 |
| ***Socioeconomic Disadvantage*** | 1.51 | 0.86 | 1.76 | 0.079 | -0.17 | 3.19 | 1.01 | 0.04 | 0.26 | 0.798 | 0.94 | 1.08 |
| ***Diagnosis Categories (Ref: No)*** |  |  |  |  |  |  |  |  |  |  |  |  |
| ***Mood Disorder*** | 2.88 | 2.20 | 1.31 | 0.191 | -1.44 | 7.21 | 1.05 | 0.10 | 0.53 | 0.596 | 0.87 | 1.27 |
| ***Psychotic Disorder*** | 7.02 | 4.74 | 1.48 | 0.14 | -2.30 | 16.33 | 1.67 | 0.35 | 2.46 | 0.014 | 1.11 | 2.51 |
| ***Substance Disorder*** | -6.66 | 2.44 | -2.73 | 0.006 | -11.45 | -1.88 | 0.85 | 0.09 | -1.47 | 0.143 | 0.69 | 1.06 |
| ***Personality Disorders*** | -6.02 | 2.77 | -2.17 | 0.03 | -11.45 | -0.58 | 0.85 | 0.10 | -1.35 | 0.176 | 0.67 | 1.08 |
| ***Anxiety Disorders*** | 5.68 | 2.84 | 2.00 | 0.046 | 0.10 | 11.26 | 1.12 | 0.14 | 0.94 | 0.346 | 0.88 | 1.43 |
| ***Self Harm/Suicide*** | -2.20 | 3.22 | -0.68 | 0.496 | -8.53 | 4.14 | 0.89 | 0.13 | -0.83 | 0.407 | 0.67 | 1.17 |
| ***Adjustment Disorder*** | -11.60 | 4.31 | -2.69 | 0.007 | -20.06 | -3.13 | 0.61 | 0.11 | -2.66 | 0.008 | 0.42 | 0.88 |
| ***Childhood Psychiatric Disorders*** | 10.28 | 8.39 | 1.22 | 0.221 | -6.20 | 26.76 | 0.96 | 0.34 | -0.12 | 0.903 | 0.48 | 1.92 |
| ***Behavioural Disorders*** | 0.19 | 3.89 | 0.05 | 0.96 | -7.44 | 7.83 | 0.97 | 0.17 | -0.15 | 0.881 | 0.69 | 1.37 |
| ***Delirium/Dementias*** | 21.83 | 8.16 | 2.68 | 0.008 | 5.81 | 37.86 | 1.66 | 0.59 | 1.43 | 0.152 | 0.83 | 3.33 |
| ***Phosphate/Oedema*** | 10.93 | 3.58 | 3.05 | 0.002 | 3.90 | 17.96 | 1.35 | 0.21 | 1.94 | 0.052 | 1.00 | 1.83 |
| ***Acute Malnutrition Markers*** | -2.18 | 2.82 | -0.77 | 0.439 | -7.71 | 3.35 | 1.27 | 0.16 | 1.98 | 0.047 | 1.00 | 1.62 |
| ***Effects of Chronic Malnutrition*** | 13.77 | 2.92 | 4.71 | <0.001 | 8.03 | 19.50 | 1.22 | 0.15 | 1.60 | 0.109 | 0.96 | 1.55 |
| ***Gastrointestinal Issues*** | 2.28 | 2.39 | 0.95 | 0.341 | -2.41 | 6.97 | 1.16 | 0.12 | 1.51 | 0.132 | 0.96 | 1.42 |
| ***Hypokalemia/Alkalosis*** | -2.14 | 3.23 | -0.66 | 0.507 | -8.48 | 4.20 | 0.85 | 0.12 | -1.14 | 0.254 | 0.65 | 1.12 |
| ***Dehydration*** | -1.67 | 3.10 | -0.54 | 0.591 | -7.76 | 4.43 | 1.04 | 0.14 | 0.29 | 0.775 | 0.79 | 1.36 |
| ***Cardiac Sequelae*** | 4.28 | 2.80 | 1.53 | 0.128 | -1.23 | 9.79 | 1.08 | 0.13 | 0.60 | 0.550 | 0.85 | 1.37 |
| ***Low Blood Pressure*** | 4.30 | 2.65 | 1.62 | 0.105 | -0.91 | 9.51 | 1.15 | 0.13 | 1.23 | 0.217 | 0.92 | 1.45 |
| ***Hypoglycemia*** | 6.77 | 2.93 | 2.31 | 0.021 | 1.00 | 12.53 | 1.08 | 0.14 | 0.62 | 0.534 | 0.85 | 1.38 |

*Wald Test for significance of multinomial variables

**Supplementary 6**. Final prediction model (multivariable linear regression) and bootstrap internal validation with 1000 bootstrap repetitions

|  | **Final Prediction Model** | | | | | | **Bootstrapped Estimates** | | | | |
| --- | --- | --- | --- | --- | --- | --- | --- | --- | --- | --- | --- |
| ***Variables*** | ***Coefficient*** | ***SE*** | ***t*** | ***p*** | ***95% CI (Lower)*** | ***95% CI (Upper)*** | ***SE*** | ***t*** | ***p*** | ***95% CI (Lower)*** | ***95% CI (Upper)*** |
| ***Diagnosis Counts*** | 1.17 | 0.21 | 5.48 | <0.001 | 0.75 | 1.59 | 0.26 | 4.45 | <0.001 | 0.66 | 1.69 |
| ***Eating Disorder Diagnoses (Ref: Anorexia Nervosa)*** | - | - | - | 0.032* | - | - | - | - | 0.001* | - | - |
| Bulimia Nervosa | -8.51 | 3.33 | -2.56 | 0.011 | -15.05 | -1.98 | 2.32 | -3.66 | <0.001 | -13.07 | -3.96 |
| Other | -3.68 | 2.88 | -1.28 | 0.202 | -9.33 | 1.97 | 1.98 | -1.86 | 0.064 | -7.56 | 0.21 |
| ***Medical Ward (Ref: No Medical Ward)*** |  |  |  |  |  |  |  |  |  |  |  |
| Medical Ward | 9.67 | 3.22 | 3.01 | 0.003 | 3.35 | 15.98 | 4.29 | 2.25 | 0.024 | 1.26 | 18.08 |
| ***Mental Health Ward (Ref: No Mental Health Ward)*** |  |  |  |  |  |  |  |  |  |  |  |
| Mental Health Ward | 19.42 | 3.53 | 5.50 | <0.001 | 12.48 | 26.36 | 4.35 | 4.46 | <0.001 | 10.89 | 27.95 |
| ***Specialist ED Ward (Ref: No Specialist ED Ward)*** |  |  |  |  |  |  |  |  |  |  |  |
| Specialist ED Ward | 56.45 | 3.36 | 16.81 | <0.001 | 49.85 | 63.04 | 4.32 | 13.06 | <0.001 | 47.97 | 64.92 |
| ***Marital Status (Ref: Not Married/De facto)*** |  |  |  |  |  |  |  |  |  |  |  |
| Married/De facto | -7.51 | 3.21 | -2.34 | 0.020 | -13.82 | -1.19 | 2.80 | -2.68 | 0.007 | -13.00 | -2.01 |
| ***Index of Relative Socioeconomic Disadvantage*** | 1.82 | 0.85 | 2.15 | 0.032 | 0.16 | 3.49 | 0.88 | 2.06 | 0.039 | 0.09 | 3.56 |
| ***Substance Disorder*** | -8.12 | 2.39 | -3.39 | 0.001 | -12.82 | -3.42 | 2.13 | -3.81 | <0.001 | -12.30 | -3.94 |
| ***Personality Disorders*** | -7.09 | 2.66 | -2.66 | 0.008 | -12.32 | -1.85 | 2.33 | -3.05 | 0.002 | -11.65 | -2.53 |
| ***Anxiety Disorders*** | 7.57 | 2.87 | 2.64 | 0.008 | 1.94 | 13.20 | 3.46 | 2.19 | 0.029 | 0.79 | 14.35 |
| ***Adjustment Disorder*** | -11.64 | 4.37 | -2.66 | 0.008 | -20.22 | -3.05 | 4.66 | -2.50 | 0.013 | -20.77 | -2.50 |
| ***Phosphate/Oedema*** | 11.30 | 3.55 | 3.18 | 0.002 | 4.33 | 18.26 | 5.03 | 2.25 | 0.025 | 1.44 | 21.15 |
| ***Effects of Chronic Malnutrition*** | 11.23 | 2.88 | 3.90 | <0.001 | 5.58 | 16.88 | 3.72 | 3.02 | 0.003 | 3.94 | 18.52 |

*Wald Test for significance of multinomial variables

**Supplementary 7.** Sensitivity Analysis, Initial exploratory linear model compared to negative binomial model

|  | **Final Prediction Linear Model** | | | | | | **Final Prediction Negative Binomial Model** | | | | | |
| --- | --- | --- | --- | --- | --- | --- | --- | --- | --- | --- | --- | --- |
| ***Variables*** | ***Coefficient*** | ***SE*** | ***t*** | ***p*** | ***95% CI (Lower)*** | ***95% CI (Upper)*** | ***IRR*** | ***SE*** | ***z*** | ***p*** | ***95% CI (Lower)*** | ***95% CI (Upper)*** |
| ***Diagnosis Counts*** | 1.17 | 0.21 | 5.48 | <0.001 | 0.75 | 1.59 | 1.06 | 0.01 | 6.10 | <0.001 | 1.04 | 1.08 |
| ***Eating Disorder Diagnoses (Ref: Anorexia Nervosa)*** | - | - | - | 0.032* | - | - |  |  |  |  |  |  |
| Bulimia Nervosa | -8.51 | 3.33 | -2.56 | 0.011 | -15.05 | -1.98 | 0.72 | 0.10 | -2.33 | 0.02 | 0.55 | 0.95 |
| Other | -3.68 | 2.88 | -1.28 | 0.202 | -9.33 | 1.97 | 0.82 | 0.10 | -1.62 | 0.104 | 0.65 | 1.04 |
| ***Medical Ward (Ref: No Medical Ward)*** |  |  |  |  |  |  |  |  |  |  |  |  |
| Medical Ward | 9.67 | 3.22 | 3.01 | 0.003 | 3.35 | 15.98 | 1.08 | 0.14 | 0.59 | 0.554 | 0.84 | 1.39 |
| ***Mental Health Ward (Ref: No Mental Health Ward)*** |  |  |  |  |  |  |  |  |  |  |  |  |
| Mental Health Ward | 19.42 | 3.53 | 5.50 | <0.001 | 12.48 | 26.36 | 2.27 | 0.35 | 5.39 | <0.001 | 1.69 | 3.06 |
| ***Specialist ED Ward (Ref: No Specialist ED Ward)*** |  |  |  |  |  |  |  |  |  |  |  |  |
| Specialist ED Ward | 56.45 | 3.36 | 16.81 | <0.001 | 49.85 | 63.04 | 6.36 | 0.91 | 12.87 | <0.001 | 4.80 | 8.43 |
| ***Marital Status (Ref: Not Married/De facto)*** |  |  |  |  |  |  |  |  |  |  |  |  |
| Married/De facto | -7.51 | 3.21 | -2.34 | 0.02 | -13.82 | -1.19 | 0.85 | 0.11 | -1.19 | 0.235 | 0.66 | 1.11 |
| ***Socioeconomic Disadvantage*** | 1.82 | 0.85 | 2.15 | 0.032 | 0.16 | 3.49 | 1.01 | 0.04 | 0.25 | 0.802 | 0.94 | 1.08 |
| ***Substance Disorder*** | -8.12 | 2.39 | -3.39 | 0.001 | -12.82 | -3.42 | 0.81 | 0.08 | -2.15 | 0.032 | 0.66 | 0.98 |
| ***Personality Disorders*** | -7.09 | 2.66 | -2.66 | 0.008 | -12.32 | -1.85 | 0.73 | 0.08 | -2.87 | 0.004 | 0.59 | 0.91 |
| ***Anxiety Disorders*** | 7.57 | 2.87 | 2.64 | 0.008 | 1.94 | 13.20 | 1.15 | 0.14 | 1.20 | 0.231 | 0.91 | 1.45 |
| ***Adjustment Disorder*** | -11.64 | 4.37 | -2.66 | 0.008 | -20.22 | -3.05 | 0.55 | 0.10 | -3.40 | 0.001 | 0.38 | 0.77 |
| ***Phosphate/Oedema*** | 11.30 | 3.55 | 3.18 | 0.002 | 4.33 | 18.26 | 1.35 | 0.20 | 2.03 | 0.042 | 1.01 | 1.79 |
| ***Effects of Chronic Malnutrition*** | 11.23 | 2.88 | 3.90 | <0.001 | 5.58 | 16.88 | 1.21 | 0.14 | 1.71 | 0.088 | 0.97 | 1.51 |
